# Heightened cholesterol 25-hydroxylase expression in aged lung during *Streptococcus pneumoniae*

**DOI:** 10.3389/fragi.2024.1480886

**Published:** 2024-12-09

**Authors:** David G. Thomas, Jianjun Yang, Soo Jung Cho, Heather Stout-Delgado

**Affiliations:** ^1^ Department of Medicine, Pulmonary and Critical Care, Weill Cornell Medicine, New York, NY, United States; ^2^ New York-Presbyterian Hospital/Weill Cornell Medicine, New York, NY, United States

**Keywords:** lipid metabolism, *Streptococcus* pneumoniae, aging, cholesterol, CH25H, macrophage

## Abstract

**Introduction:**

Alveolar macrophages (AM) are critical effectors of the immune response and are essential for host responses to *Streptococcus pneumoniae*. Changes in lipid metabolism in AM can alter cellular function and biology. Impaired metabolism can contribute to excessive lipid accumulation and pro-inflammatory signaling. Our current study was designed to examine the role of cholesterol 25-hydroxylase (Ch25h), a redox enzyme that catalyzes the oxidation of cholesterol to 25-hydroxycholesterol (25-HC), in modulating AM responses in the aged lung during *S. pneumoniae* infection.

**Methods:**

To observe the impact of aging on Ch25h expression *in* AM during infection, *in vitro* and *in vivo* murine models of *S. pneumoniae* were used.

**Results:**

At baseline and in response to infection, cholesterol metabolism significantly altered in aged AM, which corresponded with increased lipid droplet formation. *In vitro,* treatment of aged macrophages with Ch25 h-specific siRNA improved *S. pneumoniae* clearance and enhanced phagocytic receptor expression. *In vivo* siRNA targeting significantly reduced Ch25h expression in aged lungs and improved clinical parameters during *S. pneumoniae* infection. Reduction of Ch25h was associated with changes in phagocytosis and antibacterial signaling, correlated with changes in cholesterol metabolism, and increased *S. pneumoniae* clearance.

**Discussion:**

The results of our current study demonstrate that Ch25h plays an essential role in modulating aged AM responses to *S. pneumoniae*.

## Introduction

Normal lung aging is associated with multiple structural and functional changes in the respiratory tract (Quirk et al., 2016; Verbeken et al., 1992; Childs et al., 2015; Liu and Hornsby, 2007). Age-associated changes in intrinsic mechanisms that aid in cell regeneration and repair, such as depletion of adult stem cell reservoirs, mitochondrial dysfunction, increased oxidative stress, and telomere shortening, contribute to an inability of lung cells to maintain baseline homeostasis (Cho and Stout-Delgado, 2020). Acute respiratory distress syndrome (ARDS), the most severe form of acute lung injury (ALI), is a serious respiratory illness, and older patients are at a higher risk for developing this complication (Ely et al., 2002). ARDS is commonly caused by bacterial pneumonia, for which the most frequently responsible pathogen is S*treptococcus pneumoniae* (Ortqvist et al., 2005; Gotts et al., 2019). Severe pneumonia is the most common cause of ARDS but may also complicate ARDS from other causes and can contribute to prolonged respiratory failure in ARDS (Ely et al., 2002; Siner and Pisani, 2007).

Alveolar macrophages (AM) are long-lived tissue-resident innate immune cells of the airways. AMs play an essential role in mediating the host response to *S. pneumoniae*. Cellular function and biology in AM can be significantly impacted by changes in lipid metabolism. Impaired metabolism can contribute to lipid accumulation, initiation of the unfolded protein response, and induction of pro-inflammatory signaling cascades (Maxfield and Tabas, 2005; Yan and Horng, 2020). Chronic exposure to low levels of pro-inflammatory cytokines can alter the responsiveness of circulating monocytes and macrophages to pathogen-associated molecular patterns (PAMPs). Altered migratory potential can increase the recruitment of less mature myeloid cells with hyperinflammatory responses into the lung.

Cholesterol 25-hydroxylase (Ch25h) is a redox enzyme, mainly localized in the endoplasmic reticulum (ER) and Golgi apparatus, that catalyzes the oxidation of cholesterol to 25-hydroxycholesterol (25-HC) (Walther and Farese, 2012). 25-HC is an endogenous oxysterol crucial in mediating cholesterol homeostasis and regulates multiple metabolic pathways, including cholesterol synthesis, export, and esterification (Afonso et al., 2018; Goldstein et al., 2002). Heightened expression of Ch25h and 25-HC has been correlated with increased secretion of cytokines and chemokines (Liu et al., 1997; Lemaire-Ewing et al., 2009; Fu et al., 2014). Specifically, in response to virus or treatment with TLR-agonists, there is a significant Ch25h dependent upregulation of IFNR/JAK/STAT1 signal transduction pathways (Bauman et al., 2009; Diczfalusy et al., 2009; Karuna et al., 2015; Madenspacher et al., 2020; Blanc et al., 2013; Park and Scott, 2010; Zhang et al., 2018). During infection, mice with Ch25h overexpression were highly susceptible to *Listeria monocytogenes*, *Mycobacterium tuberculosis,* and influenza (Liu et al., 2011; Zou et al., 2011; Gold et al., 2014). By altering membrane cholesterol, Ch25h has been shown to play a crucial role in inhibiting pathogen entry (Zang et al., 2020; Wang et al., 2020; Yuan et al., 2019).

Ch25h, via LXR-dependent prevention of AM lipid overload, has been shown to promote efferocytosis and resolution of LPS-induced lung injury (Madenspacher et al., 2020). Models of LPS-induced acute lung injury have demonstrated a potential dose-dependent role of Ch25h and 25-HC, with increased levels of 25-HC contributing to decreased LPS-induced activation of AM (Bottemanne et al., 2021). 25-HC can decrease pro-inflammatory signaling at low concentrations, with increased signaling observed at higher 25-HC concentrations. Recent work has shown that Ch25h was essential for initiating and intensifying cytokine and chemokine production in the lung during *S. pneumoniae* infection (Cho et al., 2023). Knockdown of *Ch25h* gene expression resulted in enhanced phagocytosis and clearance of *S. pneumoniae* by alveolar macrophages (Cho et al., 2023).

Our current study was designed to investigate the impact of aging on Ch25h expression in response to *S. pneumoniae*. Using *in vitro* and *in vivo* murine models of *S. pneumoniae*, we observed an age-associated increase in Ch25h expression in aged AM during infection. At baseline and in response to infection, a significant alteration in cholesterol metabolism in aged AM corresponded with increased lipid droplet formation. *In vitro* treatment of aged macrophages with Ch25h specific siRNA improved *S. pneumoniae* clearance and enhanced phagocytic receptor expression. *In vivo,* siRNA targeting significantly reduced Ch25h expression in aged AM and improved clinical parameters during *S. pneumoniae*. *In vivo,* the reduction of Ch25h was associated with changes in phagocytosis and antibacterial signaling and was correlated with changes in cholesterol metabolism and increased *S. pneumoniae* clearance. The results of our current study demonstrate a vital role for Ch25h in modulating aged AM responses to *S. pneumoniae*.

## Materials and methods

*Mice:* Male and female wild-type young (3 months, Charles Rivers Laboratories) and aged (18+ months) (N.I.A. Rodent Colony, Charles Rivers Laboratories) BALB/c mice were housed in the Weill Cornell Medicine animal facility and handled under identical husbandry conditions and fed certified commercial feed, PicoLab Rodent Diet 20, diet 5,053 (LabDiet, Richmond, VA). The IACUC at Weill Cornell Medicine approved the use of animals in this study, and methods were carried out per the relevant guidelines and regulations. No animals were used in the study if there was evidence of skin lesions, weight loss, or lymphadenopathy.

*Human Subjects*. Adults (n = 38) aged 19 years or older with streptococcal pneumonia as diagnosed by positive airway culture (sputum, aspirates, bronchoalveolar lavage) who were admitted to New York Presbyterian Hospital, Weill Cornell Medicine were enrolled for plasma isolation obtained from whole blood, based on approval by the Human Investigational Committee. Healthy age-matched controls (n = 32) were also recruited.

*Cell Isolation and Culture*: Alveolar macrophages (AM) were isolated from freshly isolated lung tissue as previously described (Cho et al., 2023). Siglec F^+^ CD64^+^ CD11c^+^ CD11b^−^ AM populations were allowed to rest for 1 hour before each experiment. Bone marrow-derived macrophages (BMM) were generated using previously published methods (Inaba et al., 1992; Zhang et al., 2008; Cho et al., 2018b). On day 7 of culture, BMM were replated (1 × 10^6^ cells/ml) 24 h before infection or stimulation. *In vitro siRNA:* Primary macrophage cultures were transfected with 50 nM of missense or Ch25h specific (Catalog #: GS12642, Qiagen) siRNA using the GenMute siRNA Transfection Reagent for Primary Macrophages (Catalog #: SL100568-PMG, SignaGen Laboratories).

*In vivo Procedures and Tissue Collection*: *Streptococcus pneumoniae infection*: All mice were instilled intranasally with PBS or 1 × 10^3^ CFU of *S. pneumoniae* (50-μL volume in PBS) (Cho et al., 2023). *Bronchoalveolar lavage (BAL):* BAL was collected using previously published methods (Sun et al., 2017; Cho et al., 2023). *Bacterial titer assay of lung tissue*: Lung tissue samples were homogenized before serial dilution in Todd Hewitt Broth (Cho et al., 2023). *In vivo siRNA*: Ambion *In Vivo* Pre-Designed Missense and Ch25h specific siRNA (siRNA I.D. #: s63916, ThermoFisher Scientific) was complexed using the Invivofectamine 3.0 reagent (Catalog #: IVF3005, ThermoFisher Scientific) per manufacturer’s instructions. Briefly, siRNA (2.4 mg/ml) was incubated with complexation buffer (1:1 ratio) before incubation with Invivofectamine 3.0 (30 min, 50°C). siRNA complexes were diluted 6-fold with sterile PBS before instillation. Mice were instilled with siRNA 48 h before instillation with *S. pneumoniae*. *Pulse oximetry*: Vital signs and pulse oximetry were assessed using a mouse collar probe and analyzed by the Mouse Ox Plus small animal vital signs monitor (Starr Life Sciences).

*RNA Purification and Real-Time PCR*: RNA samples were extracted using the automated Maxwell RNA extraction protocol (Madison, WI, United States). Samples were reverse transcribed using the First Stand Synthesis Kit and quantified using the RT^2^ Profiler™ PCR Assays (Qiagen: Mouse Antibacterial Response, PAMM-148Z, and Mouse Phagocytosis Array, PAMM-1 3Z). In additional experiments, TaqMan probes (Catalog #: Ch25h, Mm00515486_s1 and 18sRNA, Mm03928990_g1) were purchased from ThermoFisher Scientific. Samples for TaqMan analysis were reverse transcribed using SuperScript VILO MasterMix, and expression was assessed using TaqMan Fast Advanced Master Mix (ThermoFisher Scientific). Results were quantified using Qiagen Gene Globe’s analytical software or the dCT method.

*Lipid Droplet Quantification*: Primary macrophages were stained with 2 μM of BODIPY 493/503 (Catalog #: D3922, ThermoFisher Scientific) staining solution in PBS. Images were scanned and quantified using the EVOS FL Auto Imaging System (ThermoFisher Scientific). Lipid droplet numbers were assessed in 50 random cells, and the average number was quantified.

*Cholesterol Levels*: Total lipid rafts were isolated from young and aged macrophages using the Minute Total Lipid Raft Isolation Kit for Mammalian Cells/Tissues (Catalog #: LR-039, Invent Biotechnologies Inc.) before cholesterol assessment using the Cholesterol Glo Assay (Catalog #: J3190, Promega).

*Ch25h ELISA*: Human plasma samples were analyzed for Ch25h (Catalog #: OKCD01912, Aviva Systems Biology) per the manufacturer’s instructions.

*Western Blot Analysis*: Protein was isolated from macrophages using the Minute Total Protein Extraction Kit for Animal Cultured Cells and Tissues (Catalog #: SD-001/SN-002, Invent Biotechnologies). A phosphatase/protease inhibitor cocktail (Catalog #: 5,872, Cell Signaling Technology) was added at lysis time. Protein concentration was assessed, and equal amounts of protein (20 μg/lane) were added to 4%–12% gradient gel. Immunodetection was performed using primary antibodies (Ch25h: Catalog # PA5-72349 ThermoFisher Scientific, β-actin: Catalog # 8,457 Cell Signaling Technology) at 1:1,000 dilution and secondary α-rabbit (Catalog #: 7,074, Cell Signaling Technology) at 1:2000 dilution. Images were acquired on film and analyzed using UN-SCAN-IT software.

*Ultrahigh performance liquid chromatography-tandem mass spectrometry (UPLC-MS/MS)*: Lipidomic extraction, data acquisition, and analysis were performed by Metabolon. Lipid class concentrations were calculated from the sum of all molecular species within a class, and fatty acid compositions were determined by calculating the proportion of each class comprised of individual fatty acids.

*Statistical Analysis*: Survival analysis between groups was calculated using the Mantel-Cox test. A comparison of groups was performed using a two-tailed t-test or one-way ANOVA. Bonferroni correction was used to account for multiple comparisons. Adjusted P values for each significant finding are detailed within each figure legend. All samples were independent, and statistical significance was determined for results from independent biological replicates. The sample size was at least N = 3 for *in vitro* experiments and N = 5–10 for *in vivo* experiments. All data were analyzed using GraphPad Prism software (San Diego, CA, United States). Statistical significance was considered by a *p < 0.05, **p < 0.01, ***p < 0.001, and ****p < 0.0001.

## Results

**FIGURE 1:**
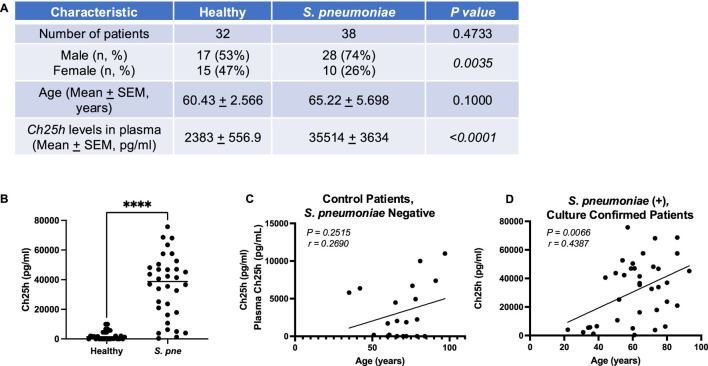
*Increased Ch25h Expression during Streptococcus pneumoniae is Associated with Age*. **(A)** Baseline characteristics and Ch25h levels in the plasma of healthy controls (n = 32) and *Streptococcus pneumoniae* patients (n = 38). The chi-square test was used to assess the statistical significance of groups. **(B)** Ch25h expression in healthy and *S. pneumoni*ae patients. Comparison of Ch25h protein expression in **(C)** control, *Streptococcus pneumoniae* negative and **(D)** *Streptococcus pneumoniae*, culture-confirmed plasma by patient age (years). P-value obtained by Mann-Whitney test. The normality of data was assessed using the D’Agostino & Pearson test. ****P < 0.0001.

Our published findings illustrate that an age-associated increase in mitochondrial and endoplasmic reticulum (ER) stress during *S. pneumoniae* infection contributed to dysregulated, overly heightened pro-inflammatory immune responses in the lung (Plataki et al., 2019; Cho et al., 2018a; Cho et al., 2018b). Specifically, using chronologically aged murine models, our findings demonstrated that decreased ATP production was associated with dysregulated mitochondrial complex expression, enhanced oxidative stress, diminished antioxidant responses, heightened activation of the UPR, and reduced numbers of healthy mitochondria in aged adult macrophages and lung in response to *S. pneumoniae* (Cho et al., 2018a; Cho et al., 2018b; Plataki et al., 2019). When compared to young adults (3 months), *S. pneumoniae* infection in aged adult (18 months) mice resulted in significantly increased bacterial titers in the lung, increased mortality, and immune cell migration/retention in lung tissue (Cho et al., 2018a; Cho et al., 2018b; Plataki et al., 2019). To better understand the factors that might contribute to this phenotype, we examined changes in oxysterol metabolism, specifically expression of Ch25h, in plasma samples collected from control and *S. pneumoniae* confirmed patients (Figure 1). We observed significantly increased Ch25h plasma expression in *S. pneumoniae*-confirmed patients (Figures 1A,B). We next examined if there was an association between age and Ch25h expression in control and *S. pneumoniae-confirmed* patient groups (Figures 1C,D). In healthy patients, there was no significant association between age and Ch25h expression (Figure 1C). In contrast, in *S. pneumoniae* confirmed patients, there was a significant association between age and Ch25h expression, with increasing age associated with higher Ch25h levels (Figure 1D).

**FIGURE 2:**
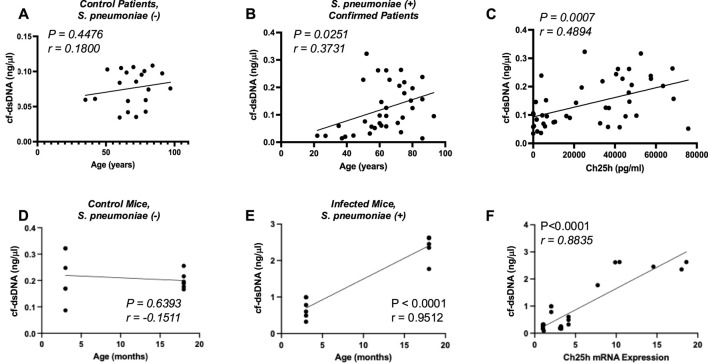
*Association of Cell-Free dsDNA with Ch25h*. **(A)** cf-dsDNA levels in control, *Streptococcus pneumoniae* is negative, and **(B)** *Streptococcus pneumoniae* culture-confirmed plasma by patient age (years). **(C)** Comparison of cf-dsDNA levels with Ch25h protein expression in control and *Streptococcus pneumoniae* positive human plasma samples. **(D)** cf-dsDNA levels present in murine plasma at 24 h post-instillation in control (PBS treated) and **(E)** *Streptococcus pneumoniae*-instilled mice by mouse age (months). **(F)** Comparison of cf-dsDNA levels with Ch25h gene expression in PBS and *Streptococcus pneumoniae* instilled murine plasma samples. P-value obtained by Mann-Whitney test. The normality of data was assessed using the D’Agostino & Pearson test.

Previous work has demonstrated that cell-free double-stranded DNA (cf-dsDNA) is a sensitive indicator and can aid in measuring disease progression (Arosemena et al., 2021; Tanaka et al., 2024; Whalen et al., 2022; Andargie et al., 2021; Erdem et al., 2024). We examined if there was an age-associated distribution of cf-dsDNA levels with patient age. There was no significant association of cf-dsDNA with age in control plasma samples (Figure 2A). In contrast, a significant association between cf-dsDNA and age was observed in plasma samples isolated from *S. pneumoniae*-positive patients (Figure 2B). We next investigated if there was an association between cf-dsDNA levels and Ch25h protein expression in plasma from control and *S. pneumoniae*-positive patients. Our results demonstrate an association between cf-dsDNA and Ch25h expression in plasma, with higher levels of cf-dsDNA correlating with increased Ch25h (Figure 2C). Based on these findings, we quantified cf-dsDNA in plasma isolated from control and *S. pneumoniae*-infected mice. While similar levels of cf-dsDNA were quantified in young and aged adult controls, a significant increase was observed in plasma isolated from mice infected with *S. pneumoniae* (Figures 2D,E). Compared to Ch25h expression in the lung, there was a significant association between increased cf-dsDNA levels and Ch25h expression (Figure 2F).

**FIGURE 3:**
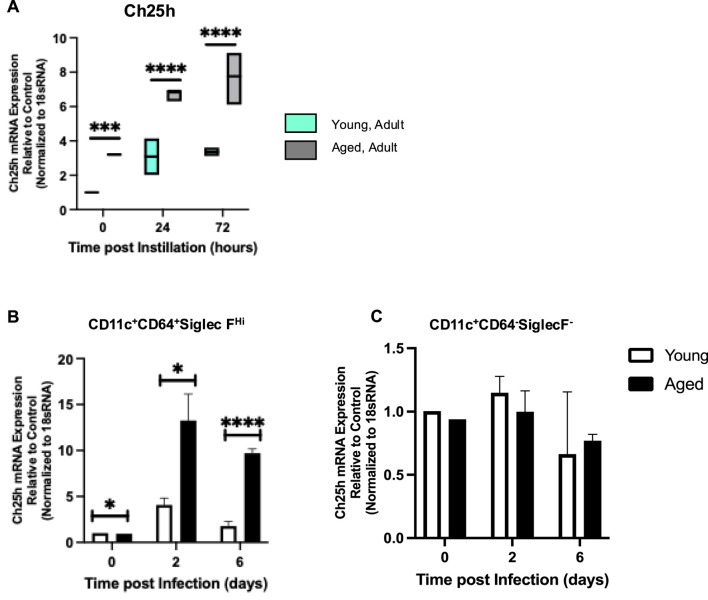
*Ch25h Mediated Production of 25-HC is Elevated in Aged Murine Lung during Streptococcus pneumoniae*. Young (3 months) and aged (18 months) mice were intranasally instilled with saline or *Streptococcus pneumoniae* (1 × 10^5^ CFU) on day 0. Lung tissue was harvested at 24- and 72-hours post-installation. **(A)** RNA was isolated from the whole control and infected lung, and *Ch25h* expression was analyzed with specific TaqMan gene assays. One-way ANOVA, P < 0.0001. Bonferroni comparison test: Y vs A PBS: ***P = 0.0003, Y vs A 24h: P < 0.0001, and Y vs A 72h: ****P < 0.0001. *Ch25h* expression was assessed in alveolar macrophage **(B)** enriched and **(C)** depleted populations isolated from young and aged lungs. N = 5–10 mice per group were used for the experiments. **(B)** One-way ANOVA, P < 0.0001. Bonferroni comparison test: Y vs A PBS: *P = 0.0248, Y vs A 24h: *P = 0.0210, Y vs A 72h: ****P < 0.0001. **(C)** One-way ANOVA, P = 0.5294. Bonferroni comparison test: Y vs A PBS: P = 0.4936, Y vs A 24h: P > 0.9999, Y vs A 72h: P > 0.9999.

To expand these results, we investigated if there was an age-associated alteration in Ch25h expression in young and aged lungs at baseline and in response to infection (Figure 3). Examination of the whole lung demonstrated a significantly increased Ch25h expression in the aged lung at baseline and in response to *S. pneumoniae* (Figure 3A). To determine the cell population contributing to Ch25h expression in the aged lung, we enriched CD64^+^CD11C^+^CD11B^−^SiglecF^Hi^ macrophages and examined changes during *S. pneumoniae* infection. Compared to young, a significant increase in Ch25h expression was observed in aged macrophages at baseline, which continued to increase in response to infection (Figure 3B). In contrast, we observed similar Ch25h expression in young and aged CD11c^+^CD64^−^SiglecF^-^ cells (Figure 3C).

**FIGURE 4:**
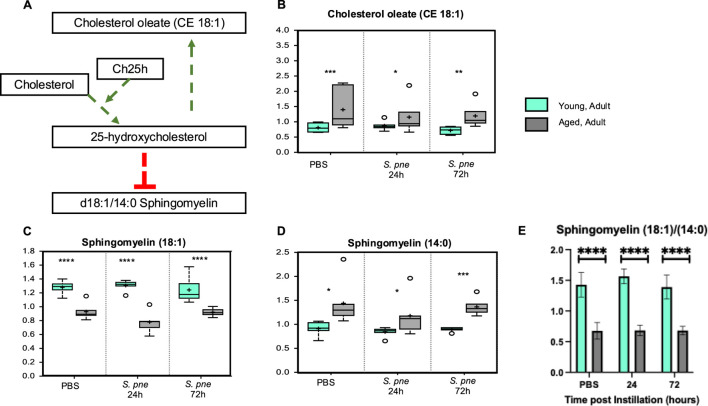
*Increased Cholesterol Oleate is Associated with Decreased Sphingomyelin (18:1/14:0) in Aged Murine Lungs during Streptococcus pneumoniae*. Young (3 months) and aged (18 months) mice were intranasally instilled with saline or *Streptococcus pneumoniae* (1 × 10^5^ CFU) on day 0. Lung tissue was harvested at 24- and 72-hours post-installation. Analysis of lipids in control and *Streptococcus pneumoniae* lung tissue was assessed by UPLC-MS. **(A)** Correlation of cholesterol oleate with 25-hydroxycholesterol and sphingomyelins. P-value of pathway overlap = 0.00113. Levels of cholesterol oleate (18:1) **(B)** One-way ANOVA, P < 0.0001. Bonferroni comparison test: Y vs A PBS: ***P = 0.0002, Y vs A 24h: *P = 0.0381, Y vs A 72h: **P = 0.0013), sphingomyelin (18:1) **(C)** One-way ANOVA, P < 0.0001. Bonferroni comparison test: Y vs A PBS: ****P < 0.0001, Y vs A 24h: ****P < 0.0001, Y vs A 72h: ****P < 0.0001), and sphingomyelin (14:0) **(D)** One-way ANOVA, P = 0.0012. Bonferroni comparison test: Y vs A PBS: *P = 0.0349, Y vs A 24h: *P = 0.0339, Y vs A 72h: ***P = 0.0006). **(E)** Sphingomyelin (18:1/14:0) ratio in young and aged lung. One-way ANOVA, P < 0.0001. Bonferroni comparison test: Y vs A PBS: ****P < 0.0001, Y vs A 24h: ****P < 0.0001, Y vs A 72h: ****P < 0.0001). N = 6 mice per group were used for the experiments.

To help identify specific lipid metabolism pathways, lipid levels were assessed using ultrahigh performance liquid chromatography-tandem mass spectroscopy (UPLC-MS/MS) with subsequent analysis using Ingenuity Pathway Analysis software (Qiagen). This secondary analysis identified 25-hydroxycholesterol (25-HC) as a significant regulator of heightened lipid metabolism in the aged lung at baseline and during *S. pneumoniae* (Figure 4A). Previous work has demonstrated cholesterol ester formation and sphingolipid formation (Shibata et al., 2013). Specifically, cholesterol oleate (CE 18:1) was significantly upregulated by 25-HC (Shibata et al., 2013). In contrast, the production of sphingomyelins, specifically the ratio of sphingomyelin 18:1 and 14:0, decreased in response to 25-HC (Shibata et al., 2013). To confirm that 25-HC upregulation was biologically significant, we analyzed the production of cholesterol oleate (CE 18:1) and sphingomyelin (18:1) and (14:0). In agreement with previous findings, our results demonstrate that in response to *S. pneumoniae*, there was a significant increase in cholesterol oleate in the aged lung (Figure 4B). We next evaluated if the sphingomyelin (18:1/14:0) ratio was altered in the aged lung. At baseline and in response to *S. pneumoniae*, there were significantly lower levels of sphingomyelin (18:1) in the aged lungs compared to the young (Figure 4C). Higher levels of sphingomyelin (14:0) were observed in the aged lungs at baseline and during infection (Figure 4D). While there were no significant changes in values for the time of infection, an age-associated difference in 18:1/14:0 ratios was observed (Figure 4E).

**FIGURE 5:**
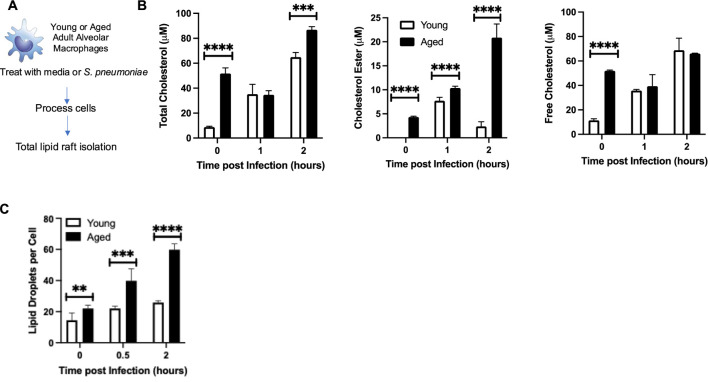
*Ch25h in Aged Alveolar Macrophages is Associated with Increased Cholesterol Levels*. **(A)** Alveolar macrophages were isolated from young (3 months) and aged (18 months) BALB/c before treatment with media alone or media containing *Streptococcus pneumoniae* (MOI 10). At select time points of infection, cells were collected and washed with PBS, and total lipid rafts were isolated. **(B)** Total cholesterol, cholesterol ester, and free cholesterol levels were assessed. *Total cholesterol*: One-way ANOVA, P < 0.0001, Bonferroni comparison test: Y vs A PBS: ****P < 0.0001, Y vs A 1h: P > 0.9999, Y vs A 2h: ***P = 0.0002. *Cholesterol ester*: One-way ANOVA, P < 0.0001, Bonferroni comparison test: Y vs A PBS: ****P < 0.0001, Y vs A 1h: ****P < 0.0001, Y vs A 2h: ****P < 0.0001. *Free cholesterol:* One-way ANOVA, P < 0.0001, Bonferroni comparison test: Y vs A PBS: ****P < 0.0001, Y vs A 1h: P > 0.9999, Y vs A 2h: P > 0.9999. **(C)** Lipid droplets per cell were quantified in 50 randomly picked young or aged macrophages. The average number of droplets is shown. One-way ANOVA, P < 0.0001, Bonferroni comparison test: Y vs A PBS: **P = 0.0016, Y vs A 0.5h: ***P = 0.0009, Y vs A 2h: ****P < 0.0001. Experiments were repeated at least three times with a N = 6 per group. Student’s t-test, ****P < 0.0001.

We examined if an age-associated increase in *Ch25h* might be attributed to changes in baseline cholesterol metabolism in aged AMs. To this extent, we enriched CD64^+^ CD11c^+^ CD11b^−^ macrophages from young and aged lungs and isolated the lipid raft population at baseline and during infection with *S. pneumoniae* (Figure 5A). Compared to the young, there was significantly higher total cholesterol, comprised of increased cholesterol esters and free cholesterol, present in lipid rafts isolated from aged macrophages at baseline and in response to infection (2 h) (Figure 5B). We observed increased cholesterol ester and free cholesterol populations in aged macrophages at baseline (Figure 5B). During infection, there was a significant increase in cholesterol ester formation quantified in aged macrophages, with similar levels of free cholesterol observed in both young and aged macrophages (Figure 5B). We next investigated if increased cholesterol ester formation might impact lipid droplet production in aged macrophages. Compared to young, in response to *S. pneumoniae*, there was a significant increase in lipid droplets in aged macrophages (Figure 5C).

**FIGURE 6:**
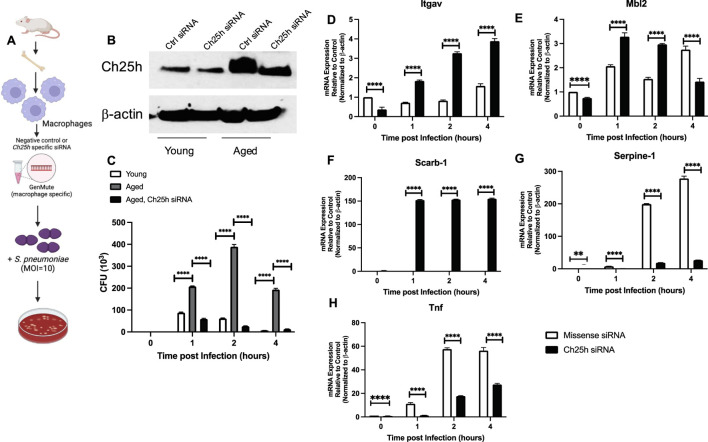
*Ch25h siRNA Treatment of Aged Macrophages Improves Streptococcus pneumoniae Clearance and Enhances Phagocytic Receptor Expression*. **(A)** Young and aged bone marrow-derived macrophages were treated with negative, missense control or Ch25h specific siRNA 24 h before infection with *Streptococcus pneumoniae* (MOI 10). **(B)** Protein was collected in siRNA-transfected macrophages to determine baseline knockdown of Ch25h (uncropped Western blot, Supplemental Figure S1). **(C)** Media was collected at select time points of culture and serially diluted to assess CFU. One-way ANOVA: P < 0.0001. Bonferroni comparison test: Y vs A 1h ****P < 0.0001, A vs A siRNA 1h ****P < 0.0001, Y vs A 2h ****P < 0.0001, A vs A siRNA 2h ****P < 0.0001, Y vs A 4h ****P < 0.0001, A vs A siRNA ****P < 0.0001. **(D–H)** Cells were collected at select time points and mRNA was isolated. Gene expression was evaluated using the Mouse Phagocytosis Array, PAMM-173Z. Specific genes, such as **(D)** *Itgav*: One-way ANOVA P < 0.0001, Bonferroni comparison test: A vs A siRNA 0h ****P < 0.0001, A vs A siRNA 1h ****P < 0.0001, A vs A siRNA 2h ****P < 0.0001, A vs A siRNA 4h ****P < 0.0001, **(E)** *Mbl2*: One-way ANOVA P < 0.0001, Bonferroni comparison test: A vs A siRNA 0h ****P < 0.0001, A vs A siRNA 1h ****P < 0.0001, A vs A siRNA 2h ****P < 0.0001, A vs A siRNA 4h ****P < 0.0001, **(F)** *Scarb-1*: One-way ANOVA P < 0.0001, Bonferroni comparison test: A vs A siRNA 0h P > 0.999, A vs A siRNA 1h ****P < 0.0001, A vs A siRNA 2h ****P < 0.0001, A vs A siRNA 4h ****P < 0.0001, **(G)** *Serpine-1*: One-way ANOVA P < 0.0001, Bonferroni comparison test: A vs A siRNA 0h **P = 0.0013, A vs A siRNA 1h ****P < 0.0001, A vs A siRNA 2h ****P < 0.0001, A vs A siRNA 4h ****P < 0.0001, and **(H)** *Tnf*: One-way ANOVA P < 0.0001, Bonferroni comparison test: A vs A siRNA 0h ****P < 0.0001, A vs A siRNA 1h **P = 0.0017, A vs A siRNA 2h ****P < 0.0001, A vs A siRNA 4h ****P < 0.0001*,* are shown. Experiments were repeated at least three times with a N = 5 per group. An overview image was generated using BioRender.

To gain better insight into the potential role of heightened Ch25h on aged macrophage responses to *S. pneumoniae*, we treated macrophages with missense or Ch25h specific siRNA and assessed bacterial clearance (Figure 6A). In response to Ch25h specific siRNA, we observed decreased expression in aged macrophages that corresponded to a significant increase in *S. pneumoniae* clearance (Figures 6B,C). We investigated the potential mechanism by which Ch25h siRNA treatment might contribute to increased *S. pneumoniae* clearance by aged macrophages. At select time points post-infection, we evaluated expression values of phagocytic receptors in aged macrophages treated with missense and Ch25h siRNA before culture with *S. pneumoniae* (Figure 6A). In response to a decrease in Ch25h expression, there was a significant increase in receptors, *Itgav* and *Mbl2* (Figures 6D,E). Ch25h siRNA treatment was also associated with a substantial increase in the expression of lipid scavenger receptors, such as *Scarb-1*, during *S. pneumoniae* (Figure 6F). We next evaluated the impact of Ch25h knockdown on inflammatory cytokines, such as *Serpine-1* and *Tnf*, and observed a significant decrease in expression in aged macrophages during infection (Figures 6G,H).

**FIGURE 7:**
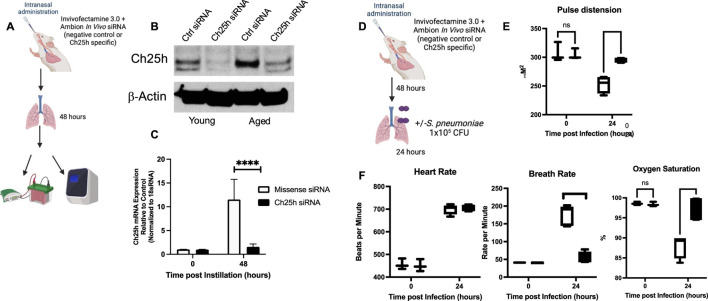
*In vivo siRNA Treatment Decreases Ch25h Expression in Aged Lung*. **(A)** Mice were intranasally instilled with missense or Ch25h specific siRNA complexed with Invivofectamine 3.0 before lung collection at 48 h post instillation. **(B)** Protein was collected from young and aged lungs at 48 h post-siRNA instillation, and Ch25h expression was assessed by Western blot. **(C)** RNA was collected from aged lungs treated with missense or Ch25h specific siRNA, and gene expression was quantified by TaqMan assay. One-way ANOVA P < 0.0001, Bonferroni comparison test: A vs A siRNA 0h P > 0.999, A vs A siRNA 48h ****P < 0.0001. **(D)** Aged BALB/c mice were intranasally instilled with missense or Ch25h specific siRNA 48 h before infection with *Streptococcus pneumoniae* (1 × 10^4^ CFU). **(E–F)** Pulse distension: One-way ANOVA P < 0.0001, Bonferroni comparison test: A vs A siRNA 0h P > 0.999, A vs A siRNA 24h ****P < 0.0001, heart rate: One-way ANOVA P < 0.0001, Bonferroni comparison test: A vs A siRNA 0h P > 0.999, A vs A siRNA 24 h P > 0.999, breath rate: One-way ANOVA P < 0.0001, Bonferroni comparison test: A vs A siRNA 0h P > 0.999, A vs A siRNA 24h ****P < 0.0001, and oxygen saturation: One-way ANOVA P < 0.0001, Bonferroni comparison test: A vs A siRNA 0h P > 0.999, A vs A siRNA 24h ***P = 0.0002 were assessed in missense, and Ch25h siRNA was treated in aged mice at baseline and in response to infection. N = 5–10 mice per group were used for the experiments. To confirm these findings, experiments were repeated at least three times. Experimental overview images were generated using BioRender.

Based on these findings, we investigated the potential role of Ch25h on innate immune responses in aged lungs during *S. pneumoniae*. For these experiments, we utilized an *in vivo* specific siRNA formulation and highly purified locked nucleic acid (LNA)-modified 21-bp siRNA duplex with overhangs to minimize the innate immune responses to siRNA com lex. Mice were instilled with control (missense) or Ch25h specific siRNA, and lung tissue was harvested 48 h post-instillation (Figure 7A). In response to Ch25h siRNA, a significant decrease in Ch25h protein and mRNA expression was detected in aged lungs (Figures 7B,C). To determine if *in vivo* treatment with Ch25h siRNA might alter physiological responses, we measured alterations in heart rate, breath rate, and oxygen saturation at baseline and in response to *S. pneumoniae* (Figure 7D). While no significant difference was detected at baseline, we observed a substantial improvement in pulse distension, breath rate, and oxygen saturation in aged Ch25h siRNA-treated lungs during *S. pneumoniae* infection (Figures 7E,F).

**FIGURE 8:**
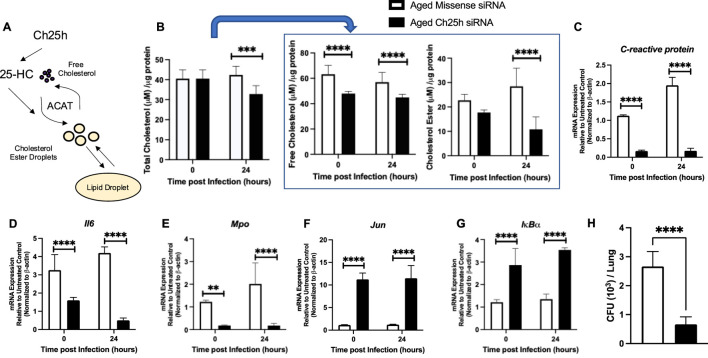
*In vivo Treatment with Ch25h siRNA Reduces Cholesterol Ester Droplets in Aged Lung at Baseline and during Streptococcus pneumoniae*. **(A)** Role of Ch25h in the metabolism of free cholesterol and cholesterol ester droplets. **(B)** Lung tissue was collected and processed using the Cholesterol Glo Assay kit. Samples were serially diluted and incubated with or without esterase. Total cholesterol: One-way ANOVA *p* = 0.0003, Bonferroni comparison test: A vs A siRNA 0h P > 0.999, A vs A siRNA 24h ***P = 0.0003, free cholesterol: One-way ANOVA P < 0.0001, Bonferroni comparison test: A vs A siRNA 0h ****P < 0.0001, A vs A siRNA 24h ****P < 0.0001, and cholesterol esters: One-way ANOVA P < 0.0001, Bonferroni comparison test: A vs A siRNA 0h P > 0.999, A vs A siRNA 24h ****P < 0.0001 were quantified in missense and Ch25h siRNA-treated lung at baseline and in response to *Streptococcus pneumoniae*. **(C–G)** RNA was isolated from the lung, and gene expression was assessed using the Mouse Antibacterial Response, PAMM-148Z. Changes in the expression of **(C)** *C-reactive protein:* One-way ANOVA P < 0.0001, Bonferroni comparison test: A vs A siRNA 0h ****P < 0.0001, A vs A siRNA 24h ****P < 0.0001, **(D)** *Il-6:* One-way ANOVA P < 0.0001, Bonferroni comparison test: A vs A siRNA 0h ****P < 0.0001, A vs A siRNA 24h ****P < 0.0001, **(E)** *myeloperoxidase:* One-way ANOVA P < 0.0001, Bonferroni comparison test: A vs A siRNA 0h **P = 0.0057, A vs A siRNA 24h ****P < 0.0001, **(F)** *Jun:* One-way ANOVA P < 0.0001, Bonferroni comparison test: A vs A siRNA 0h ****P < 0.0001, A vs A siRNA 24h ****P < 0.0001, and **(G)** *IκBα:* One-way ANOVA P < 0.0001, Bonferroni comparison test: A vs A siRNA 0h ****P < 0.0001, A vs A siRNA 24h ****P < 0.0001 are shown. **(H)** Lung homogenates were serially diluted, and CFU was quantified. Student’s t-test, P < 0.0001. N = 5–10 mice per group were used for the experiments. To confirm these findings, experiments were repeated at least three times. Experimental overview images were generated using BioRender.

We next evaluated a potential mechanism by which treatment of aged lung with Ch25h siRNA might improve host lung function during *S. pneumoniae*. Given the role of Ch25h derived 25-HC in modulating the production of cholesterol esters, we examined the impact of siRNA treatment on cholesterol levels at baseline and in response to infection (Figure 8A). Treatment with Ch25h specific siRNA reduced total cholesterol levels in lung tissue in response to *S. pneumoniae* (Figure 8B). We observed significantly lower free cholesterol levels in both missense and Ch25h specific siRNA treated lung and a significant reduction in cholesterol ester formation detected in Ch25h siRNA treated lung (Figure 8B). We next evaluated the potential impact of Ch25h siRNA treatment on antibacterial signaling in the lung at 24 h post-infection. Compared to missense-treated lungs, a significant decrease in *C-reactive protein*, *Il-6*, and *myeloperoxidase* gene expression was observed in aged lungs treated with Ch25h siRNA at baseline and during *S. pneumoniae* (Figures 8C–E). We also observed a significant increase in *c-Jun* and *IκBα* expression in aged Ch25h siRNA-treated lungs associated with improved bacterial clearance (Figures 8F–H).

Our current study demonstrates the essential role of Ch25h in modulating immune responses in the aged lung during *S. pneumoniae*.

## Discussion

Our current study was designed to investigate the impact of aging on Ch25h expression in response to *S. pneumoniae*. Using *in vitro* and *in vivo* murine models of *S. pneumoniae*, we observed an age-associated increase in Ch25h expression in aged AM during infection. At baseline and in response to infection, a significant alteration in cholesterol metabolism in aged AM corresponded with increased lipid droplet formation. *In vitro* treatment of aged macrophages with Ch25h specific siRNA improved *S. pneumoniae* clearance and enhanced phagocytic receptor expression. *In vivo,* the reduction of Ch25h was associated with changes in phagocytosis and antibacterial signaling and was correlated with changes in cholesterol metabolism and increased *S. pneumoniae* clearance. Our current study demonstrates that enhanced Ch25h expression in aged AM and lung can detrimentally impact host responses to *S. pneumoniae*.

Previous work has illustrated that 25-HC can regulate IL-6 secretion by macrophages and epithelial cells (Gold et al., 2014; Koarai et al., 2012). In agreement with these studies, reduced Ch25h post-treatment with Ch25h siRNA resulted in significantly decreased levels of IL-6 production in the aged lung during *S. pneumoniae* infection. It has been postulated that 25-HC modulates NF-kB signaling changes (Gold et al., 2014; Koarai et al., 2012). Interestingly, we observed increased levels of AP-1 transcription family member, c-Jun, in the aged lung in response to Ch25h specific siRNA treatment. Binding to the AP-1 motif by c-Jun/c-Jun homodimers can stimulate AP-1 activity and contribute to cellular signaling. Significantly, AP-1 activation can induce c-Jun mediated binding and repression of nuclear remodeling complexes, resulting in a shift from c-Jun homodimers to more stable and transcriptionally active c-Fos/c-Jun heterodimers (Ogawa et al., 2004; Papavassiliou and Musti, 2020; Aguilera et al., 2011). Recent work has also demonstrated a role for c-Jun in mediating the stress response induced by pore-forming toxins (Moyano et al., 2018). Exposure to pore-forming toxins, such as staphylococcal α-toxin, enhanced c-Jun transcripts and protein regulation levels and was associated with increased epithelial cell survival (Moyano et al., 2018). It is, therefore, possible that changes in c-Jun expression in aged lungs might significantly impact innate signaling in response to *S. pneumoniae*. Future work must be performed to fully characterize the role by which Ch25h modulates these responses.

Previous work has demonstrated that the expression of Ch25h and the production of 25-HC were significantly enhanced in the lung tissue of COPD patients (Sugiura et al., 2012). Specifically, levels of 25-HC were significantly correlated with the degree of airflow limitation, which may contribute to subepithelial fibrosis and alveolar wall destruction (Busse et al., 1999). Additional studies have demonstrated that 25-hydroxycholesterol enhances cytokine release and toll-like receptor responses in airway epithelial cells (Koarai et al., 2012). In agreement with these studies, the results of our current work suggest that enhanced Ch25h expression could enhance inflammation and lung tissue injury during *S. pneumoniae*.

The results of our current study demonstrate that early in the response to *S. pneumoniae,* an age-associated increase in Ch25h contributed to dysregulated cholesterol metabolism and increased inflammation in the murine lung. Upstream of Ch25h and 25-HC is 3β-hydroxysterol Δ^24^-reductase (Dhcr24), the enzyme that catalyzes the conversion of desmosterol to cholesterol. Overexpression of Dhcr24 has been shown to decrease desmosterol accumulation in mitochondria, increase ROS production, and contribute to inflammatory signaling (Zhang et al., 2021). In addition, molecular analysis of macrophages isolated from atherosclerotic lesions revealed that depletion of desmosterol by Dhcr24 overexpression increased the expression of genes associated with ‘classical’ macrophage activation (Zhang et al., 2021). Recent work has illustrated that in response to LPS stimulation, young adult Dhcr24 overexpressing transgenic murine macrophages do not exhibit enhanced levels of 25-HC (Zhang et al., 2021). These findings demonstrate that enhanced levels of Dhcr24 within young macrophages do not alter oxysterol content at baseline or in response to LPS activation (Zhang et al., 2021). Despite these findings, the role of Dhcr24 and other cholesterol modulators on Ch25h and cholesterol metabolism in aged lungs has not been fully elucidated.

Increased cholesterol levels can contribute to increased lung injury, as observed in ventilator-induced lung injury, idiopathic pulmonary fibrosis (IPF), and acute respiratory distress syndrome (ARDS) (Vockeroth et al., 2010; Fireman et al., 1996; Markart et al., 2007). Recent work to map the plasma lipidome during sepsis due to community-acquired pneumonia (CAP) found strong lipid class-specific associations with disease severity with triacylglycerols, cholesterol esters (CE), and lysophospholipids (Chouchane et al., 2024). A time-dependent and disease-specific shift in the plasma lipidome correlated with disease severity, systemic inflammation, and higher patient mortality (Chouchane et al., 2024). Cholesterol is essential in maintaining lung homeostasis; however, enhanced levels, potentially due to alterations in CE formation, may contribute to increased lung injury. Our findings demonstrate an age-associated increase in Ch25h detected in plasma collected from *S. pneumoniae*-confirmed patients. Ch25h expression and function may indicate disease severity, with increased expression in older persons associated with changes in cholesterol metabolism and CE formation. Future studies to investigate a potential correlation of Ch25h expression with specific lipid class associations will need to be performed to help disentangle this relationship.

Results of our current study demonstrate that early in the response to *S. pneumoniae,* an age-associated increase in Ch25h contributed to dysregulated cholesterol metabolism and increased inflammation in murine lung. It is important to note that the time points selected for siRNA-mediated knockdown of Ch25h were based on preliminary studies investigating efficacy at 24- and 48-h post-installation. Given the complex landscape of the lung, the time of siRNA delivery and subsequent infection with *S. pneumoniae* can significantly influence the results. Future studies will need to be performed to determine how additional time points may contribute to differential cell responses in the lung and the role of these on siRNA efficacy and immune responses.

It is important to note that limited human sample data was used for our study. The current study did not assess the impact of Ch25h expression on morbidity and mortality. Another limitation of our current study is that while cf-dsDNA has been previously shown to be a sensitive indicator of disease progression, multiple sources of cf-dsDNA may contribute to the relative levels detected in plasma samples. Specifically, cf-dsDNA can reflect increased host injury by releasing mitochondrial or genomic dsDNA (Tanaka et al., 2024). Importantly, circulating microbial cell-free DNA can also serve as an additional source of cf-dsDNA. Increased cf-dsDNA in plasma samples may indicate cellular injury and/or enhanced presence of microbial pathogens. In this context, cf-dsDNA would serve as an indicator of disease progression, but the source of origination would need to be identified. Future work will need to be performed using established next-generation sequencing to determine the cell and tissue of origination and the pathogenic species present.

Results of our current study demonstrate that alveolar macrophages are a source of Ch25h expression in aged lungs (Bauman et al., 2009; Diczfalusy et al., 2009). Previous reports have shown that Ch25h-dependent secretion of 25-HC occurs in macrophages in response to (Lippa et al., 2022) activation or during chronic lung diseases (Bauman et al., 2009; Sugiura et al., 2012). Notably, 25-HC can also be non-enzymatically generated by cholesterol autoxidation (Johnson et al., 1994). While the experimental focus of this study was to examine the role of Ch25h on host responses to *S. pneumoniae*, heightened 25-HC detected at baseline might be attributed to increased oxidative stress-mediated cholesterol autoxidation in the aged lung. Future work will need to be performed to determine these parameters.

## Data Availability

The raw data supporting the conclusions of this article will be made available by the authors, without undue reservation.
