# Supplementary figures and images for: Heightened cholesterol 25-hydroxylase expression in aged lung during Streptococcus pneumoniae
Source: Front Aging. 2024 Dec 9;5:1480886. doi: 10.3389/fragi.2024.1480886 (PMC11663934; doi:10.3389/fragi.2024.1480886)

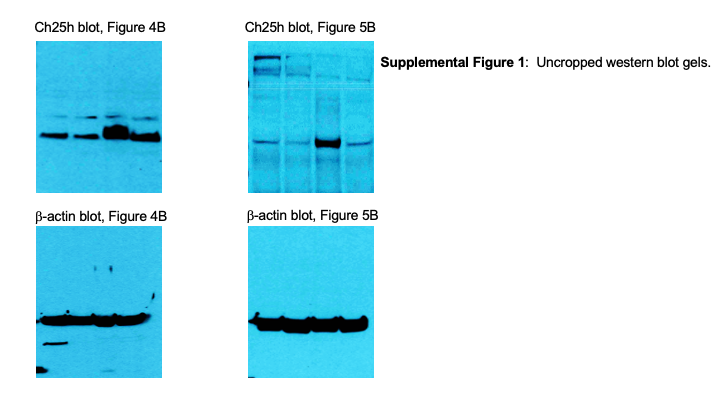

Supplement: Supplementary file 1 [file Image1.tiff]
